# Synthetic Bilirubin‐Based Nanomedicine Protects Against Renal Ischemia/Reperfusion Injury Through Antioxidant and Immune‐Modulating Activity

**DOI:** 10.1002/adhm.202403846

**Published:** 2025-01-23

**Authors:** Ji‐Jing Yan, Hyunjin Kim, Bomin Kim, Honglin Piao, Joon Young Jang, Tae Kyeom Kang, Wook‐Bin Lee, Dohyeon Kim, Seunghyun Jo, Duckhyang Shin, Sharif MD Abuzar, Myung L. Kim, Jaeseok Yang, Sangyong Jon

**Affiliations:** ^1^ Division of Nephrology Department of Internal Medicine Yonsei University College of Medicine Seoul 03722 Republic of Korea; ^2^ The Research Institute for Transplantation Yonsei University College of Medicine Seoul 03722 Republic of Korea; ^3^ BILIX.Co., Ltd. Yongin Gyeonggi‐do 16942 Republic of Korea; ^4^ Natural Product Research Center Korea Institute of Science & Technology Gangneung Gangwon‐do 25451 Republic of Korea; ^5^ Department of Biological Sciences KAIST Institute for the BioCentury Center for Precision Bio‐Nanomedicine Korea Advanced Institute of Science and Technology Daejeon 34141 Republic of Korea

**Keywords:** bilirubin nanoparticle, immune modulation, ischemia/reperfusion injury, kidney, reactive oxygen species

## Abstract

Renal ischemia/reperfusion injury (IRI) is a common form of acute kidney injury. The basic mechanism underlying renal IRI is acute inflammation, where oxidative stress plays an important role. Although bilirubin exhibits potent reactive oxygen species (ROS)‐scavenging properties, its clinical application is hindered by problems associated with solubility, stability, and toxicity. In this study, BX‐001N, a synthetic polyethylene glycol‐conjugated bilirubin 3α nanoparticle is developed and assessed its renoprotective effects in renal IRI. Intravenous administration of BX‐001N led to increase uptake in the kidneys with minimal migration to the brain after IRI. Peri‐IRI BX‐001N administration improves renal function and attenuates renal tissue injury and tubular apoptosis to a greater extent than free bilirubin on day 1 after IRI. BX‐001N suppressed renal infiltration of inflammatory cells and reduced expression of TNF‐α and MCP‐1. Furthermore, BX‐001N increases renal tubular regeneration on day 3 and suppresses renal fibrosis on day 28. BX‐001N decreases the renal expressions of dihydroethidium, malondialdehyde, and nitrotyrosine after IRI. In conclusion, BX‐001N, the first Good Manufacturing Practice‐grade synthetic bilirubin‐based nanomedicine attenuates acute renal injury and chronic fibrosis by suppressing ROS generation and inflammation after IRI. It shows adequate safety profiles and holds promise as a new therapy for renal IRI.

## Graphical abstract

BX‐001N, a polyethylene glycol‐conjugated bilirubin 3α nanoparticle, is the first GMP‐grade, synthetic bilirubin‐based nanomedicine. It effectively attenuates acute renal injury, facilitates subacute renal recovery, and suppresses chronic fibrosis after renal ischemia/reperfusion injury via anti‐oxidant and immune‐modulating activity. Therefore, BX‐001N with renoprotective effects and an adequate safety profile is a promising new therapeutic agent for renal ischemia/reperfusion injury.

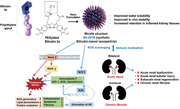

## 1 Introduction

Renal ischemia/reperfusion injury (IRI) is a common complication after renal ischemic insult. Acute injury in renal IRI is frequently accompanied by chronic renal fibrosis.^[^ ^1^, ^2^, ^3^, ^4^ ^]^ However, no definite treatment exists for renal IRI except supportive care with correction of the underlying ischemic events. The basic mechanism of renal IRI is acute inflammation where oxidative stress plays an important role.^[^ ^5^, ^6^, ^7^, ^8^ ^]^ Renal IRI induces oxidative stress and suppresses anti‐oxidant activity, while reactive oxygen species (ROS) induce renal inflammation and tissue damage.^[^ ^6^, ^7^, ^8^, ^9^ ^]^ Therefore, ROS and inflammation are potential targets for therapeutic intervention in renal IRI.

Bilirubin, a lipid‐soluble waste product that is the end product of heme catabolism, can exert damage at high concentrations, including kernicterus with neurologic complications.^[^ ^10^, ^11^ ^]^ However, bilirubin is an endogenous anti‐oxidant, exhibiting cytoprotective and anti‐inflammatory effects via its anti‐oxidant properties.^[^ ^10^, ^12^, ^13^, ^14^ ^]^ Unconjugated, free bilirubin (BR) attenuates IRI by suppressing ROS in the ex‐vivo isolated, perfused rat kidney.^[^ ^15^ ^]^ Despite potent ROS‐scavenging effects of BR, its clinical application as a therapeutic agent is limited by water‐insolubility, lack of cellular uptake due to albumin binding, oxidation‐associated instability, and requirement of the parenteral route.^[^ ^12^, ^13^, ^14^ ^]^

We previously developed the world‐first bilirubin‐based nanomedicines, including polyethylene glycol‐conjugated (PEGylated) bilirubin and its self‐assembled nanoparticles to overcome the current limitations of BR. These bilirubin nanoparticles have better safety profiles than BR and effectively suppress various ROS‐mediated diseases, including cancer and chronic inflammatory diseases.^[^ ^12^, ^13^ ^]^ Bilirubin nanoparticles also suppress acute inflammation, including IRI in the heart and liver.^[^ ^16^, ^17^ ^]^ Furthermore, bilirubin nanoparticles suppress pulmonary fibrosis, a sequela of acute and chronic inflammation.^[^ ^18^ ^]^

However, our previous forms of bilirubin nanoparticles and commercially available BR are sourced from porcine blood or bile acids, which raises concerns about infection and supply limitations, thereby hindering their clinical application.^[^ ^19^ ^]^ Recently, we succeeded in the chemical synthesis of bilirubin to address these concerns and produced the first synthetic bilirubin‐based nanoparticle, called “BX‐001N” (Brixelle) (PCT/KR2022/01 1910 filed October 8, 2022). Although BR exists as bilirubin 4α in human blood, BX‐001N consists of a chemically synthesized isomer, bilirubin‐3α, which has greater photostability and thermal stability than bilirubin 4α.^[^ ^20^, ^21^ ^]^ Furthermore, in contrast to the previous mixed form of bilirubin 3α, 9α, and 13α nanoparticles, BX‐001N is a homogenous, PEGylated bilirubin 3α nanoparticle that self‐assembles into highly uniform nanoparticles in aqueous medium.^[^ ^12^ ^]^ Based on these advancements, BX‐001N is currently under a phase 1 clinical trial (ClinicalTrials.gov ID: NCT06097702) as the only clinically applicable, Good Manufacturing Practice (GMP)‐grade bilirubin nanomedicine.

Herein, we investigated whether BX‐001N could suppress acute inflammation and subsequent chronic fibrosis after renal IRI and assessed its therapeutic potential for clinical application.

## 2 Results

### 2.1 Structure and Characterization of BX‐001N

**Figure 1:**
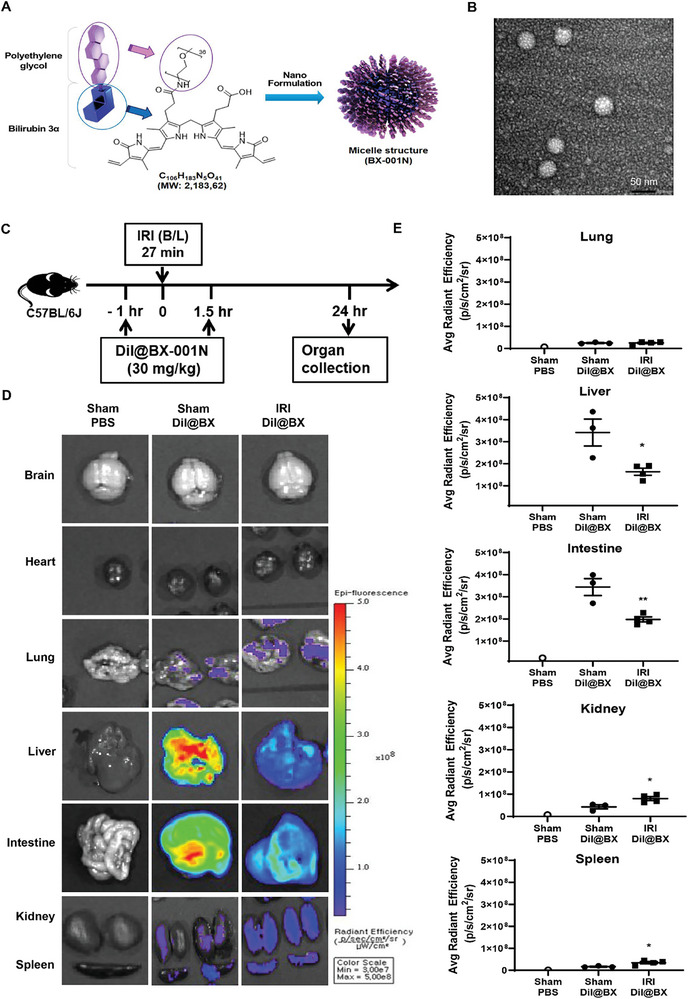
Structure and in vivo biodistribution of BX‐001N. A) BX‐001N is a self‐assembled PEGylated bilirubin 3α nanoparticle and consists of a synthetic bilirubin 3α molecule covalently bonded to 36 ethylene oxide moieties. B) The morphology of BX‐001N, as observed using transmission electron microscopy. Scale bars, 100 µm. C) Experimental scheme of BX‐001N biodistribution after IRI. D–E) Biodistribution images D) and average radiant efficiency E) of fluorescent dye Dil‐labeled BX‐001N (Dil@BX) in various organs after IRI. All samples in each group are displayed as individual dots, and lines and whiskers in dot plots indicate the mean and SEM, respectively. *p < 0.05, **p < 0.01 in comparison between the sham/Dil@BX‐001N and IRI/Dil@BX‐001N groups (Student's *t*‐test). Avg, average; B/L, bilateral; Dil, 1,1′‐Dioctadecyl‐3,3,3′,3′‐tetramethylindocarbocyanine; IRI, ischemia/reperfusion injury; MW, molecular weight; PBS, phosphate‐buffered saline; SEM, standard error of the mean.

BX‐001N (Bilix Co., Ltd., Korea) is a self‐assembled PEGylated bilirubin 3α nanoparticle and consists of a synthetic bilirubin 3α molecule covalently bonded to 36 ethylene oxide moieties (**Figure** **1**A).^[^ ^22^ ^]^ In the in vivo experiments, we administered 30 mg kg^−1^ of BX‐001N, where bilirubin content was 26.8%, equating to an equivalent dose of 8.04 mg kg^−1^ of BR (Cayman, 0496184‐1, USA). Mono‐PEGylated bilirubin 3α is amphiphilic (soluble in both aqueous and organic solvents), and its thermodynamic solubility at 25 °C in water measured using high‐performance liquid chromatography (Waters, Milford, MA, USA) was 125.7 ± 6.2 mg mL^−1^ (mean ± the standard error of the mean [SEM]). According to the United States Pharmacopeia solubility criteria, a substance with a solubility greater than 125 mg mL^−1^ is considered “freely soluble,” thereby classifying BX‐001N as a freely soluble compound. Characterization using a dynamic light scattering with zetasizer (Malvern Panalytical, EA, Netherlands) revealed that BX‐001N has a particle size of 42.44 ± 0.11 mm and polydispersity index of 0.19. Transmission electron microscopy (TEM; Tecnai G2 spirit twin FEI, Netherlands) further confirmed the morphology of BX‐001N, as shown in Figure 1B.

### 2.2 In Vivo Distribution of BX‐001N

We injected 1,1′‐Dioctadecyl‐3,3,3′,3′‐tetramethylindocarbocyanine (Dil)‐labeled BX‐001N 1 h before and 1.5 h after renal IRI to evaluate its biodistribution (Figure 1C). In the sham group, BX‐001N was mainly distributed in the liver and intestines. In contrast, in the IRI group, BX‐001N accumulated more in the kidneys and spleen and less in the liver and intestine, compared to that in the sham group (*p* < 0.05, Figure 1D,E). Notably, the brain showed a minimal fluorescent signal of BX‐001N in both the IRI and sham groups (Figure 1D,E).

### 2.3 Beneficial Effects of BX‐001N in Neutrophil‐Like Cell‐ and Macrophage‐Mediated Oxidative Stress and Inflammation

**Figure 2:**
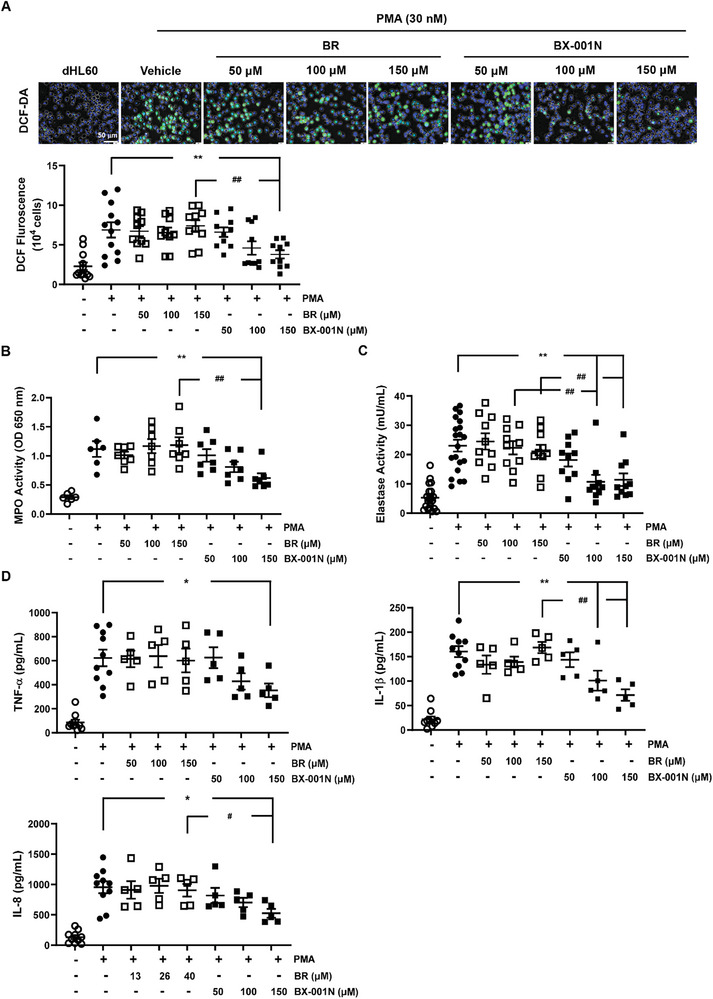
Beneficial effects of BX‐001N on neutrophil‐like cell‐mediated oxidative stress and inflammation. A) Intracellular levels of DCF‐DA in dHL‐60 cells. Green and blue indicate DCF‐DA and Hoechst, respectively. Fluorescent images at 40× magnification. B) MPO activity in dHL‐60 cells. C) Elastase activity in dHL‐60 cells. D) Protein expression levels of TNF‐α, IL‐1β, and IL‐8 in dHL‐60 cells. All samples in each group are displayed as individual dots, and lines and whiskers in dot plots indicate the mean and SEM, respectively. *p < 0.05, **p < 0.01 compared with the PMA control group (Student's *t*‐test or Mann–Whitney test). ^#^ *p* < 0.05, ^##^ *p* < 0.01 in comparison between the BX‐001N and BR groups (Student's *t*‐test or Mann–Whitney test). BR, free bilirubin group; DCF‐DA, 2′,7′‐dichlorodihydrofluorescein diacetate; dHL‐60, differentiated HL‐60; IL‐1, interleukin‐1; IL‐8, interleukin‐8; MPO, myeloperoxidase; PMA, phorbol 12‐myristate 13‐acetate; SEM, standard error of the mean; TNF‐α, tumor necrosis factor‐ α.

As ROS and innate immune cells, such as neutrophils and macrophages, are key contributors to IRI, the in vitro effects of BX‐001N on ROS generation and inflammatory responses of innate immune cells were initially assessed. BX‐001N significantly suppressed intracellular ROS (2′,7′‐dichlorodihydrofluorescein diacetate [DCF‐DA]) in differentiated HL‐60 (dHL‐60) cells compared to the control group, especially at 150 µM (*p* < 0.01, **Figure** **2**A). BX‐001N also inhibited myeloperoxidase (MPO) activity within granules, thereby suppressing oxidative bursts compared to the control (*p* < 0.01, Figure 2B). Regarding neutrophil extracellular traps (NETs), BX‐001N treatment significantly suppressed NETosis‐released elastase activity (*p* < 0.01, Figure 2C). Further supporting its immune‐modulating effects, BX‐001N reduced the secretion of pro‐inflammatory cytokines or chemokines, including tumor necrosis factor (TNF)‐α, interleukin (IL)‐1β, and IL‐8 in dHL‐60 cells (Figure 2D). In contrast, treatment with BR did not alter intracellular ROS levels or the expression levels of pro‐inflammatory cytokines and chemokines (Figure 2A–D).

**Figure 3:**
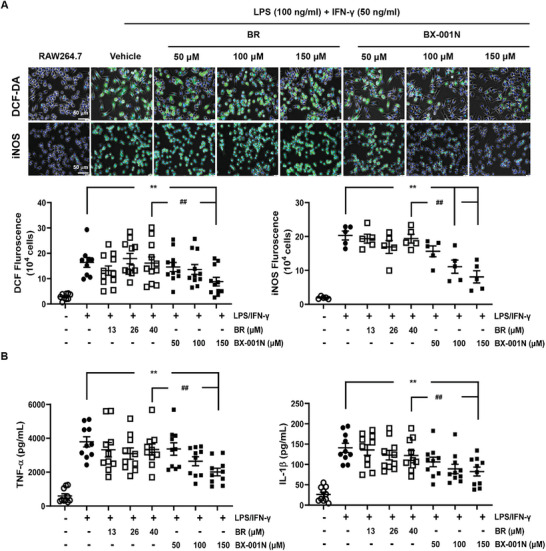
Beneficial effects of BX‐001N on macrophage‐mediated oxidative stress and inflammation. A) Intracellular levels of DCF‐DA and iNOS in RAW264.7 cells. Green and blue indicate DCF‐DA/iNOS and Hoechst, respectively. Fluorescent images at 40× magnification. B) Protein expression levels of TNF‐α and IL‐1β in RAW264.7 cells. All samples in each group are displayed as individual dots, and lines and whiskers in dot plots indicate the mean and SEM, respectively. *p < 0.05, **p < 0.01 compared with the LPS/IFN‐γ control group (Student's *t*‐test). ^#^ *p* < 0.05, ^##^ *p* < 0.01 in comparison between the BX‐001N and BR groups (Student's *t*‐test). BR, free bilirubin group; DCF‐DA, 2′,7′‐dichlorodihydrofluorescein diacetate; IL‐1, interleukin‐1; INF‐γ, interferone‐gamma; iNOS, inducible nitric oxide synthase; LPS, lipopolysaccharide; SEM, standard error of the mean; TNF‐α, tumor necrosis factor‐1.

In macrophages, BX‐001N significantly inhibited intracellular ROS production and inducible nitric oxide synthase (iNOS) expression in macrophages at concentrations of 150 µm (**Figure** **3**A). Additionally, BX‐001N suppressed the secretion of pro‐inflammatory cytokines, including TNF‐α and IL‐1β (Figure 3B). Furthermore, the BX‐001N group showed better efficacy in modulating both ROS and pro‐inflammatory cytokine levels than the BR group, which exhibited no significant modulatory effects (Figure 3A,B).

### 2.4 Cytoprotective Effect of BX‐001N on Renal Tubular Cells Against Oxidative Stress

BX‐001N treatment significantly enhanced cell viability of HK‐2 cells against the H_2_O_2_‐induced oxidative stress in a dose‐dependent manner and its potency was ≈5‐fold higher than that of BR (Figure S1A, Supporting Information). The extracellular ROS scavenging efficacy of BX‐001N demonstrated a half‐maximal effective concentration (EC_50_) of ≈50 µm, comparable to that of BR for both H₂O₂ and O₂⁻ (Figure S1B,D, Supporting Information). These findings indicate that the ROS scavenging potency of BX‐001N is largely attributed to the bilirubin structure contained within the compound. The intracellular ROS scavenging efficacy of BX‐001N exhibited an EC_50_ of ≈90 µm, while BR showed no measurable reactivity toward intracellular ROS (not calculated) (Figure S1F, Supporting Information). The ROS scavenging efficacy of BX‐001N in the hypoxia‐reoxygenation system showed similar results observed in the H₂O₂‐induced oxidative stress environment. The extracellular ROS scavenging efficacy of BX‐001N against both H_2_O_2_ and O_2_^−^ was comparable to that of BR, whereas only BX‐001N effectively scavenged intracellular ROS (H_2_O_2_, Figure S1C; O_2_^−^, Figure S1E; DCF, Figure S1G, Supporting Information). These results suggest that, unlike BR, BX‐001N is capable of scavenging both extracellular and intracellular ROS through cellular uptake. Overall, the results highlight the potential of BX‐001N as a cytoprotective agent to mitigate the detrimental effects of oxidative stress in renal cells.

### 2.5 BX‐001N Attenuated Renal Injury at Acute Phase After IRI

**Figure 4:**
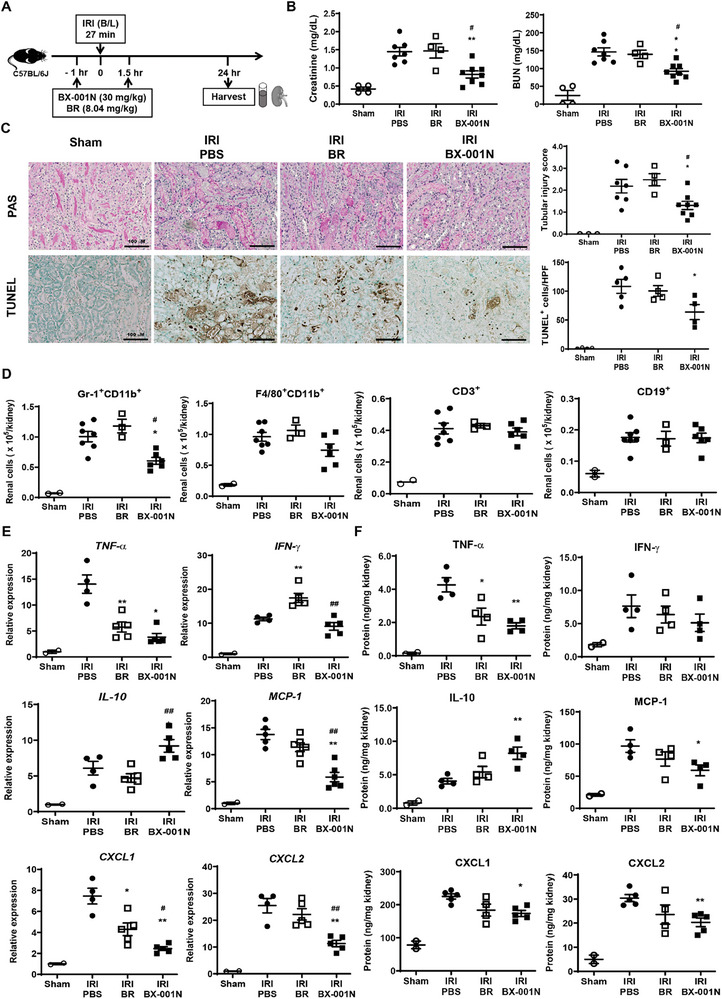
BX‐001N attenuated renal injury at acute phase after IRI. A) BX‐001N, BR, or PBS was administered to mice 1 h before and 1.5 h following renal IRI. The kidneys, along with blood samples were procured on day 1 after IRI. B) Blood levels of creatinine and BUN. C) Renal tissue injury scores (based on PAS staining) and renal tubular apoptosis (based on TUNEL staining). Scale bars, 100 µm. Magnification, 200×. D) Absolute number of renal Gr‐1^+^CD11b^+^ neutrophils, F4/80^+^CD11b^+^ macrophages, CD3^+^ T cells, and CD19^+^ B cells. E) Renal mRNA expression levels of *Tnfa*, *Ifng*, *Il10, Mcp1*, *Cxcl1*, and *Cxcl2* normalized to *Gapdh* expression levels. F) Protein expression levels of TNF‐α, INF‐γ, IL‐10, MCP‐1, CXCL1, and CXCL2. All samples in each group are displayed as individual dots, and lines and whiskers in dot plots indicate the mean and SEM, respectively. *p < 0.05, **p < 0.01 compared to the PBS group (Student's t‐test or Mann–Whitney test). ^#^ *p* < 0.05, ^##^ *p* < 0.01 in comparison between the BX‐001N and BR groups (Student's *t*‐test). BR, free bilirubin group; BUN, blood urea nitrogen; CXCL, CXC motif chemokine ligand; *Gapdh*, glyceraldehyde 3‐phosphate dehydrogenase; HPF, high‐power field; INF‐γ, interferon‐γ; IL, interleukin; IRI, ischemia/reperfusion injury; MCP‐1, monocyte chemoattractant protein‐1; PAS, periodic acid–Schiff; PBS, phosphate‐buffered saline; SEM, standard error of the mean; TNF‐α, tumor necrosis factor‐α; TUNEL, terminal deoxynucleotidyl transferase dUTP nick‐end labeling.

BX‐001N was administered 1 h before and 1.5 h after renal bilateral IRI, and kidney tissues and peripheral blood were procured 24 h after IRI to assess its effects on acute injury (**Figures** **4**A and S2A, Supporting Information). Dose‐dependent evaluations showed that renal function improvements were comparable across all doses of BX‐001N (Figure S2B, Supporting Information). However, when the renal tubular injury scores were compared, a dose‐response relationship was observed wherein higher doses provided better protection (Figure S2C, Supporting Information). We selected two doses of 30 mg kg^−1^ in the following experiments due to potential toxicity concerns with a cumulative dose exceeding 60 mg kg^−1^ in toxicity studies (data not shown). Our previous unpublished pharmacokinetic experiment in ICR mice revealed that a single 30 mg kg^−1^ injection of BX‐001N yielded maximal plasma and blood concentrations of 0.56 and 0.28 mg mL^−1^, respectively, suggesting that in vivo blood concentration of BX‐001N after one or two injections of 30 mg kg^−1^ would be similar to in vitro concentration of 128 (125–150) µm of BX‐001N.

The blood levels of creatinine and blood urea nitrogen (BUN) were significantly lower in the BX‐001N group than in the phosphate‐buffered saline (PBS) and BR groups (*p* < 0.05), whereas no significant difference was found between the BR and PBS groups (Figure 4B). Furthermore, the tubular injury score and renal tubular apoptosis as measured by terminal deoxynucleotidyl transferase‐mediated digoxigenin‐deoxyuridine nick‐end labeling (TUNEL) were significantly attenuated in the BX‐001N group compared with those in the PBS control group (*p* < 0.05); however, BR did not decrease tubular injury or apoptosis (Figure 4C). Similarly, the renal tubular expression of pro‐apoptotic markers, such as cleaved caspase‐3, bax, and cytochrome c decreased, whereas that of anti‐apoptotic markers, including bcl‐2 increased in the BX‐001N group (Figure S3, Supporting Information). In addition to reducing apoptosis, BX‐001N significantly decreased the renal infiltration of innate immune cells, such as Gr‐1^+^CD11b^+^ neutrophils compared to that in the PBS group (*p* < 0.05, Figure 4D). Furthermore, the renal expressions of TNF‐α, monocyte chemotactic protein (MCP)‐1, chemokine C‐X‐C motif ligand 1 (CXCL1), and CXCL2 in both mRNA (Figure 4E) and protein levels (Figure 4F) were suppressed in the BX‐001N group. In contrast, IL‐10 levels were higher in the BX‐001N group (Figure 4E,F). The BR group, however, showed no significant differences compared to the PBS group, except for the TNF‐α expression (Figure 4E,F).

### 2.6 BX‐001N Facilitated Renal Recovery at Subacute Phase After IRI

**Figure 5:**
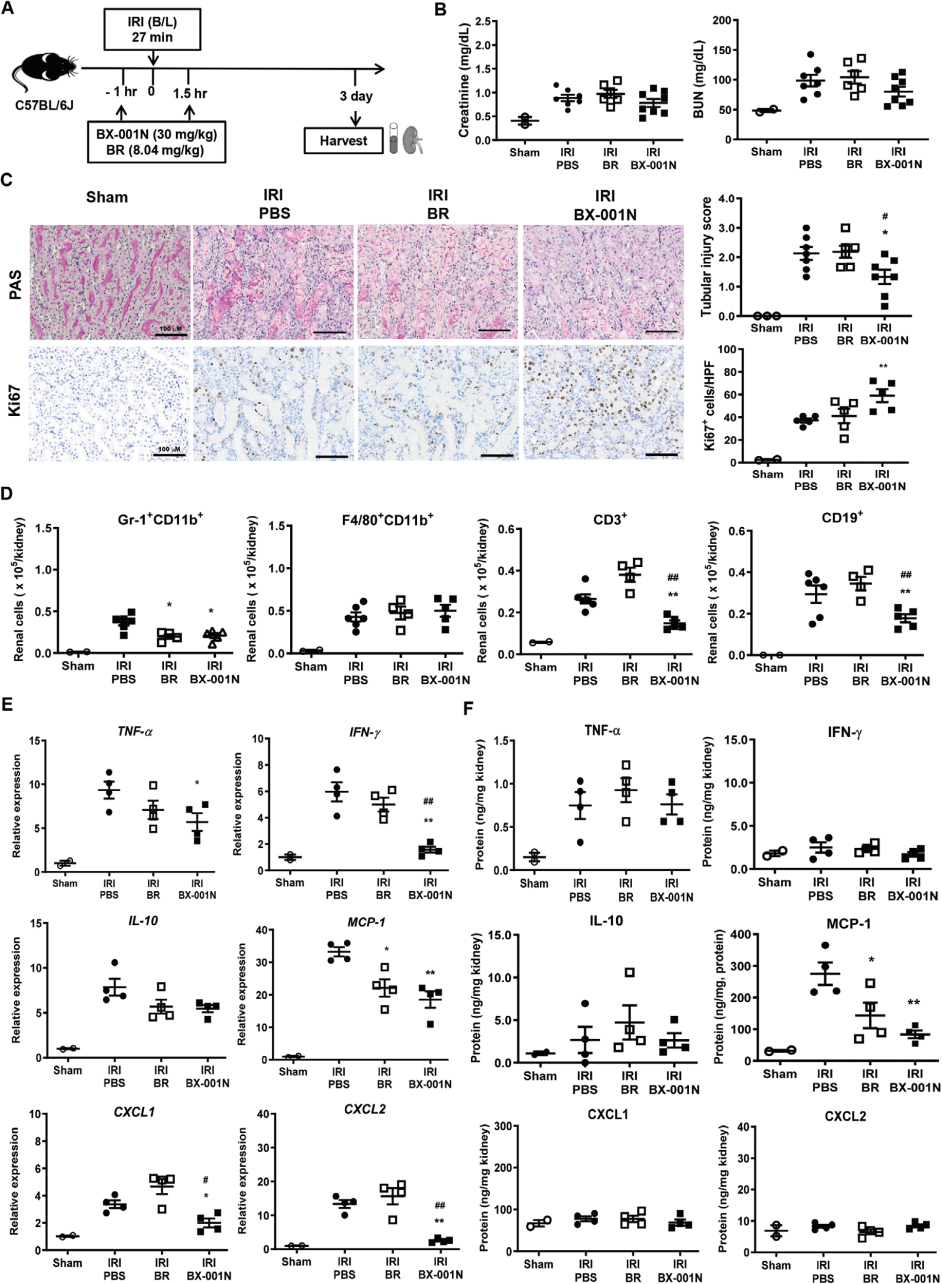
BX‐001N facilitated renal recovery at the subacute phase after IRI. A) BX‐001N, BR, or PBS was administered to mice 1 h prior to and 1.5 h following renal IRI. The kidneys along with blood samples were procured on day 3 after IRI. B) Blood levels of creatinine and BUN. C) Renal tissue injury scores (based on PAS staining) and renal tubular regeneration (Ki67 staining). Scale bars, 100 µm. Magnification, 200×. D) Absolute number of renal Gr‐1^+^CD11b^+^ neutrophils, F4/80^+^CD11b^+^ macrophages, CD3^+^ T cells, and CD19^+^ B cells. E) Renal mRNA expression levels of *Tnfa*, *Ifng*, *Il10, Mcp1*, *Cxcl1*, and *Cxcl2* normalized to *Gapdh* expression levels. F) Protein expression levels of TNF‐α, INF‐γ, IL‐10, MCP‐1, CXCL1, and CXCL2. All samples in each group are displayed as individual dots, and lines and whiskers in dot plots indicate the mean and SEM, respectively. *p < 0.05, **p < 0.01 compared to the PBS group (Student's *t*‐test). ^#^ *p* < 0.05, ^##^ *p* < 0.01 in comparison between the BX‐001N and BR groups (Student's *t*‐test or Mann–Whitney test). BR, free bilirubin group; BUN, blood urea nitrogen; CXCL, CXC motif chemokine ligand; *Gapdh*, glyceraldehyde 3‐phosphate dehydrogenase; HPF, high‐power field; INF‐γ, interferon‐γ; IL, interleukin; IRI, ischemia/reperfusion injury; MCP‐1, monocyte chemoattractant protein‐1; PAS, periodic acid–Schiff; PBS, phosphate‐buffered saline; SEM, standard error of the mean; TNF‐α, tumor necrosis factor‐α.

Kidney tissue and peripheral blood were procured 3 days after IRI to assess the effects of BX‐001N on the subacute phase (**Figure** **5**A). While the blood levels of creatinine and BUN were not significantly lower in the BX‐001N group on day 3 (Figure 5B), the tubular injury score remained significantly lower in the BX‐001N group than in the PBS and BR groups (*p* < 0.05, Figure 5C). Furthermore, renal regeneration, assessed by Ki67 staining was significantly increased in the BX‐001N group compared to that in the PBS group (*p* < 0.01, Figure 5C), indicating enhanced cellular proliferation and repair processes facilitated by BX‐001N treatment. Additionally, the renal infiltrations of CD3^+^ T cells, CD19^+^ B cells, and neutrophils in the BX‐001N group decreased compared to those in the PBS and BR groups (Figure 5D). The renal mRNA expression of TNF‐α, IFN‐γ, MCP‐1, CXCL1, and CXCL2 (Figure 5E) were suppressed in the BX‐001N group. The protein levels of MCP‐1 were similarly reduced (Figure 5F), further underscoring BX‐001N's ability to attenuate subacute inflammatory responses.

### 2.7 BX‐001N Suppressed Renal Fibrosis at Chronic Phase After IRI

**Figure 6:**
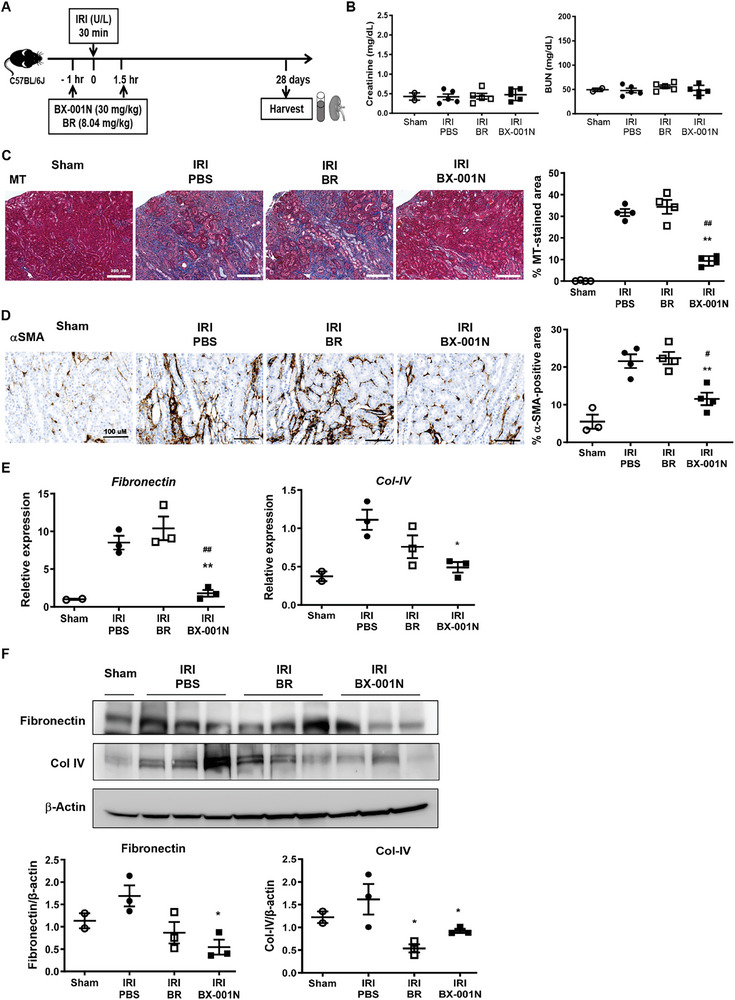
BX‐001N suppressed renal fibrosis at chronic phase after IRI. A) BX‐001N, BR, or PBS was administered to mice 1 h prior to and 1.5 h following renal IRI. Kidneys along with blood samples were procured on day 28 after IRI. B) Blood levels of creatinine and BUN. C) Renal fibrosis based on MT staining. Scale bars, 200 µm. Magnification, 100×. D) Immunohistochemical staining images for αSMA. Scale bars, 100 µm. Magnification, 200×. E) Renal mRNA expression level of *fibronectin (Fn1)* and *Col‐IV (Col4a1)* normalized to *Gapdh* expression level. F) Protein expression levels of fibronectin, Col‐IV, and β‐actin. All samples in each group are displayed as individual dots, and lines and whiskers in dot plots indicate the mean and SEM, respectively. *p < 0.05, **p < 0.01 compared to the PBS group (Student's *t*‐test). ^##^ *p* < 0.01 in comparison between the BX‐001N and BR groups (Student's *t*‐test). αSMA, α‐smooth muscle actin; BR, free bilirubin group; BUN, blood urea nitrogen; Col‐IV, type IV collagen; *Gapdh*, glyceraldehyde 3‐phosphate dehydrogenase; HPF, high‐power field; IRI, ischemia/reperfusion injury; MT, Masson's trichrome; PBS, phosphate‐buffered saline; SEM, standard error of the mean.

To evaluate the long‐term effects of BX‐001N, kidney tissue and peripheral blood were procured 28 days after IRI (**Figure** **6**A). The blood levels of creatinine and BUN were similar between the BX‐001N and PBS groups (Figure 6B). Masson's trichrome staining, however, revealed significantly reduced renal fibrosis in the BX‐001N‐treated group compared to the PBS group (*p* < 0.05, Figure 6C). Additionally, the renal expression of α‐smooth muscle actin (αSMA) was also lower in the BX‐001N group than in the PBS and BR groups (Figure 6D). Furthermore, the mRNA (Figure 6E) and protein (Figures 6F and S4A, Supporting Information) levels of key fibrotic markers, including fibronectin and type IV collagen were lower in the BX‐001N group.

### 2.8 BX‐001N Attenuated IRI‐Induced Oxidative Stress in Kidney Tissues

**Figure 7:**
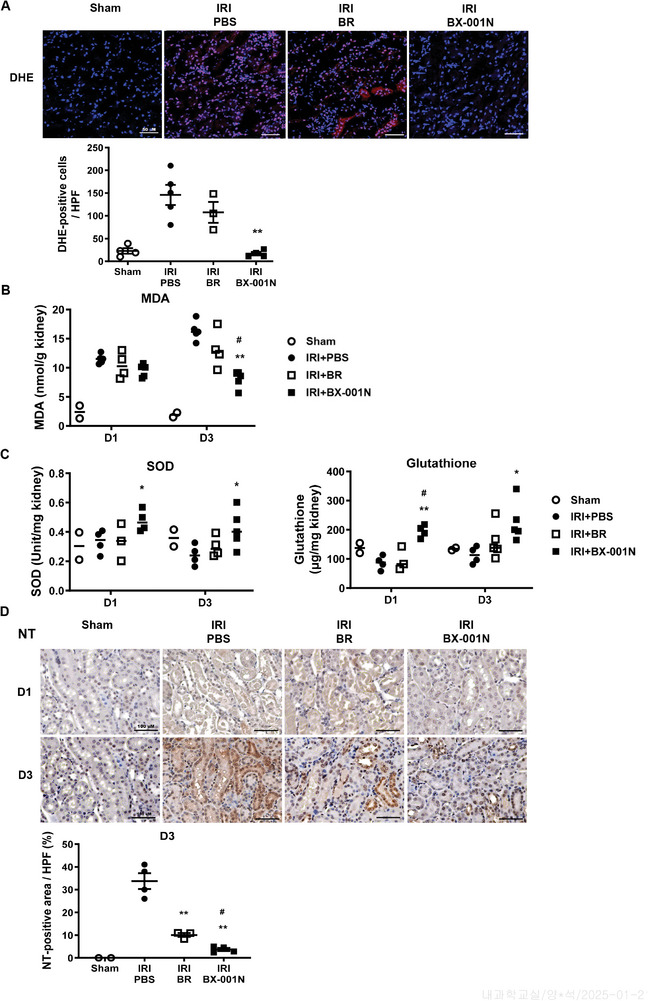
BX‐001N attenuated IRI‐induced oxidative stress in kidney tissues. A) Confocal images for DHE (red) and DAPI (blue) on day 1. Scale bars, 50 µm. Magnification, 400×. B) MDA levels in kidney tissues. C) SOD activities and glutathione levels in kidney tissues on days 1 and 3. D) Immunohistochemical staining images for nitrotyrosine in kidney tissues on days 1 and 3. Scale bars, 100 µm. Magnification, ×200. All samples in each group are displayed as individual dots, and lines and whiskers in dot plots indicate the mean and SEM, respectively. *p < 0.05, **p < 0.01 compared with the PBS group (Student's *t*‐test). ^#^ *p* < 0.05, ^##^ *p* < 0.01 in comparison between the BX‐001N and BR groups (Student's *t*‐test). BR, free bilirubin group; D1, day 1; D3, day 3; DAPI, 4′,6‐diamidino‐2‐phenylindole; DHE, dihydroethidium; HPF, high‐power field; IRI, ischemia/reperfusion injury; MDA, malondialdehyde; NT, nitrotyrosine; PBS, phosphate‐buffered saline; SEM, standard error of the mean; SOD, superoxide dismutase.

We assessed the protective effects of BX‐001N against ROS‐mediated renal injury following IRI. The dihydroethidium (DHE) staining on day 1 demonstrated that the ROS levels in kidney tissues were attenuated in the BX‐001N group (**Figure** **7**A). Additionally, BX‐001N treatment decreased renal malondialdehyde (MDA) levels (Figure 7B), whereas it increased both superoxide dismutase (SOD) activity and glutathione levels (Figure 7C). Furthermore, BX‐001N attenuated nitrotyrosine levels in kidney tissues after IRI (Figure 7D).

**Figure 8:**
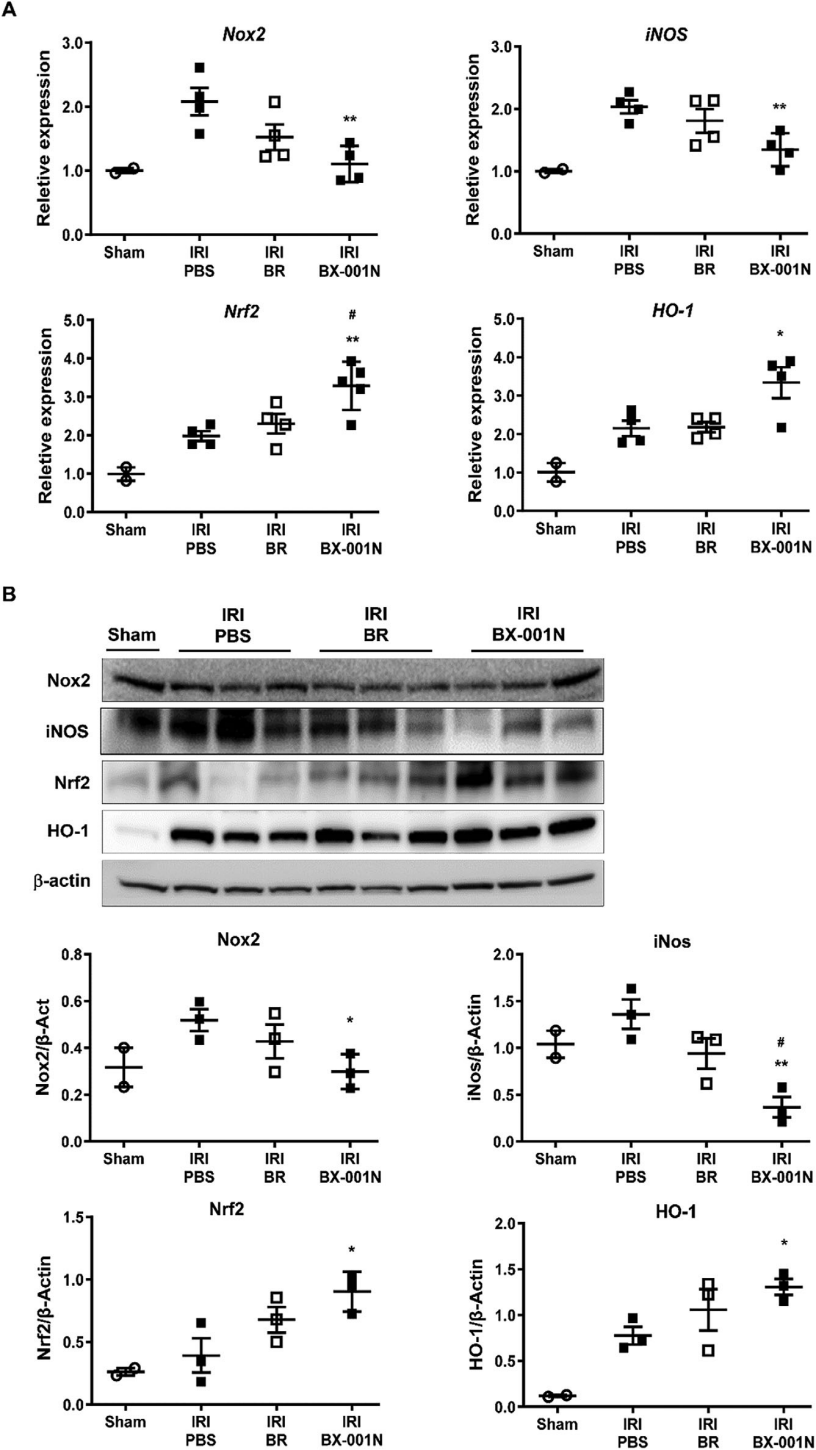
BX‐001N suppressed renal Nox2/iNOS expression and activated renal Nrf2/HO‐1 expression after IRI. A) Renal mRNA expression levels of *Nox2, iNos, Nrf2, and HO‐1* normalized to *Gapdh* expression levels on day 1 after IRI. B) Western blot images for renal expression of Nox2, iNOS, Nrf2, HO‐1, and β‐actin on day 1 after IRI. All samples in each group are displayed as individual dots, and lines and whiskers in dot plots indicate the mean and SEM, respectively. *p < 0.05, **p < 0.01 compared with the PBS group (Student's *t*‐test). ^#^ *p* < 0.05, ^##^ *p* < 0.01 in comparison between the BX‐001N and BR groups (Student's *t*‐test). BR, free bilirubin group; HO‐1, heme oxygenase‐1; iNOS, inducible nitric oxide synthase; IRI, ischemia/reperfusion injury; Nrf2, nuclear factor erythroid‐2‐related factor 2; Nox, nicotinamide adenine dinucleotide phosphate oxidase; PBS, phosphate‐buffered saline; SEM, standard error of the mean.

When the renal expressions of mediators and protectors of oxidative stress were assessed on day 1 after IRI, BX‐001N suppressed the renal expression of pro‐oxidative mediators, including Nox2 and iNOS compared to the PBS control at both the mRNA (**Figure** **8**A) and protein levels (Figures 8B and S4B, Supporting Information). In contrast, BX‐001N increased significantly upregulated the expression of key antioxidant regulators, including nuclear factor erythroid 2‐related factor 2 (Nrf2) and heme oxygenase 1 (HO‐1) at both mRNA (Figure 8A) and protein levels (Figures 8B and S4B, Supporting Information).

## 3 Discussion and Conclusion

This study demonstrated the therapeutic potential of BX‐001N, a synthetic PEGylated bilirubin 3α nanoparticle, in mitigating renal IRI by accumulating preferentially in the kidneys post‐IRI and significantly attenuating acute renal inflammation and tissue injury to a greater extent than BR. Furthermore, BX‐001N facilitated subacute renal regeneration and suppressed chronic renal fibrosis following renal IRI. The underlying mechanisms include the suppression of pro‐oxidative mediators, such as Nox2 and iNOS, and the upregulation of antioxidant regulators, including Nrf2 and HO‐1. These molecular modulations culminated in the inhibition of ROS‐mediated renal injury.

A key aspect of this study was the evaluation of BX‐001N's ROS‐scavenging and immunomodulatory potential against innate immune cells, particularly neutrophils and macrophages, which play pivotal roles in the acute inflammatory response following IRI. The in vitro experiments demonstrated that BR exhibited limited impact on intracellular ROS levels and pro‐inflammatory cytokine production. Despite being a potent antioxidant, bilirubin's inability to enter immune cells hinders its effectiveness in regulating immune activation. In contrast, BX‐001N overcame this limitation, demonstrating its ability to effectively modulate the pro‐inflammatory functions of neutrophils and macrophages. Similarly, BX‐001N effectively scavenged intracellular ROS in renal tubular epithelial cells and showed 5‐fold greater cytoprotective effects compared to BR.

BX‐001N administration improved renal functions in the acute phase following renal IRI. In parallel with in vitro protective effects of BX‐001N against ROS injury in renal tubular epithelial cells, its treatment attenuated acute renal tissue injury and renal tubular apoptosis. Furthermore, BX‐001N reduced the renal infiltration of neutrophils and macrophages with the suppression of renal expressions of TNF‐α, MCP‐1, CXCL1, and CXCL2 in the acute phase after IRI. Interestingly, BX‐001N upregulated the expression of IL‐10 with anti‐inflammatory and anti‐apoptotic properties during the acute phase.^[^ ^23^ ^]^ The upregulation of IL‐10 may result from bilirubin reductase A, which converts biliverdin to bilirubin and enhances IL‐10 expression in macrophages following IRI.^[^ ^24^ ^]^ BX‐001N likely induced IL‐10 upregulation in abundant inflammatory cells within renal tissues during the acute phase, thereby contributing to the attenuation of acute renal injury and subsequent renal recovery. However, the renoprotective effects of BR were not significant, in contrast to a previous *ex vivo* study in isolated, perfused rat kidney.^[^ ^15^ ^]^ Beyond the acute phase, BX‐001N continued to exert beneficial effects in the subacute phase by suppressing renal inflammation and facilitating renal regeneration following IRI. BX‐001N suppressed the renal infiltration of adaptive immune cells, including T and B cells, and innate immune cells, such as neutrophils, along with the downregulation of pro‐inflammatory cytokines and chemokines. The suppressive effects of BX‐001N on renal infiltration of innate and adaptive immune cells appeared to align with their infiltration kinetics into the kidneys. The suppressive effects on the infiltration of innate immune cells were most prominent during the acute phase, while those on adaptive immune cells became more marked during the subacute phase.

A complete recovery from acute kidney injury (AKI) is uncommon, and it frequently leads to chronic kidney disease (CKD).^[^ ^2^, ^3^, ^4^, ^25^, ^26^ ^]^ Acute‐to‐chronic progression, commonly referred to as AKI‐to‐CKD transition, is also common after renal IRI.^[^ ^1^, ^3^ ^]^ The AKI after IRI is frequently accompanied by chronic fibrosis in the renal tissue. A previous study involving the mixed form of bilirubin nanoparticles demonstrated the suppression of pulmonary inflammation and fibrosis within two weeks following bleomycin instillation, suggesting that BX‐001N, a homogenous form, could suppress chronic fibrosis following acute inflammation.^[^ ^18^ ^]^ Using murine renal unilateral IRI model, a well‐suited model for studying acute‐to‐chronic transition, we assessed the impact of early BX‐001N treatment on chronic phase following IRI.^[^ ^27^ ^]^ BX‐001N effectively suppressed chronic renal fibrosis, suggesting a potential role of BX‐001N in mitigating CKD progression after IRI. While previous studies primarily focused only on the acute effects of bilirubin nanoparticles in cardiac and hepatic IRI, this study comprehensively assessed BX‐001N's impact across all phases of renal IRI—acute, subacute, and chronic. The results underscore the beneficial effects of BX‐001N in renal recovery and chronic fibrosis, as well as the suppression of acute inflammation and injury. Therefore, this study provides a novel insight into the therapeutic potential of BX‐001N as a clinically applicable bilirubin nanomedicine for renal IRI.

Oxidative stress plays a pivotal role in the pathogenesis of renal IRI, generating excessive ROS via neutrophils and macrophages, and leading to inflammatory responses, lipid peroxidation (MDA), protein oxidation (nitrotyrosine), and ultimately cellular apoptosis.^[^ ^28^, ^29^, ^30^, ^31^ ^]^ Our findings revealed elevated levels of DHE, MDA, and nitrotyrosine in renal tissues after IRI. BX‐001N treatment significantly suppressed these markers in renal tissues, highlighting its potent ROS‐scavenging activity, which is a key mechanism underlying its renal protection against IRI. Additionally, BX‐001N improved the activity of crucial endogenous antioxidant enzymes, including SOD and glutathione. The enhanced activity of these endogenous antioxidant enzymes protects against oxidative stress and contributes to protection against tubular epithelial cell apoptosis.

Nox family proteins play an important role in ROS generation in the kidney.^[^ ^7^, ^13^, ^32^, ^33^ ^]^ Among the various forms of NOS that produce nitric oxide and increase oxidative stress, iNOS is upregulated by inflammation.^[^ ^7^ ^]^ Conversely, Nrf2 serves as a protective molecule against ROS.^[^ ^34^, ^35^ ^]^ HO‐1, a key downstream molecule of Nrf2^[^ ^14^, ^35^ ^]^ catabolizes heme into carbon monoxide and bilirubin, thereby exerting anti‐inflammatory and anti‐apoptotic functions.^[^ ^7^, ^36^, ^37^ ^]^ The mechanisms of antioxidant activity of bilirubin involve the suppression of TNF‐α‐induced Nox expression, inhibition of Nox‐mediated iNOS induction, and promotion of Nrf2 activation and HO‐1 upregulation.^[^ ^14^, ^38^, ^39^, ^40^, ^41^, ^42^, ^43^ ^]^ Notably, the Nrf2/HO‐1 pathway has been identified as a potential therapeutic target for renal fibrosis.^[^ ^44^ ^]^ Consistent with these findings, BX‐001N suppressed the expressions of Nox2 and iNOS while inducing those of Nrf2 and HO‐1 after IRI, suggesting that these mechanisms likely contribute to BX‐001N‐mediated renal protection in the acute and chronic phases of IRI.

BX‐001N, a synthetic PEGylated bilirubin 3α nanoparticle, showed considerably better in vitro ROS scavenging and anti‐inflammatory effects than an equivalent dose of BR. Furthermore, BX‐001N exhibited good in vivo renoprotective effects against renal IRI, whereas BR exhibited minimal in vivo renoprotective effects. These superior effects of BX‐001N over BR could be attributed to several factors.^[^ ^14^ ^]^ First, the hydrophilic PEGylation of BX‐001N enhances its water solubility under physiological conditions, overcoming a major limitation of BR. Second, PEGylation of BX‐001N improves the in vivo stability of bilirubin, prevents oxidation, and prolongs its half‐life.^[^ ^14^ ^]^ The clearance of BX‐001N in rats was ≈ 20–30‐fold lower (0.33–0.67 mL min^−1^ kg^−1^) than the published clearance of BR (12.8 mL min^−1^ kg^−1^), indicating a low systemic clearance of BX‐001N compared to slowly‐metabolized and ‐eliminated BR.^[^ ^22^, ^45^ ^]^ Third, BX‐001N efficiently localizes to inflamed target organs, thereby minimizing off‐target side effects.^[^ ^17^, ^46^ ^]^ This study also showed increased retention of BX‐001N in the kidneys and spleen after IRI, which are rich in inflammatory cells and ROS.^[^ ^14^ ^]^

Neurotoxicity is a well‐recognized adverse effect of bilirubin therapy, primarily linked to high systemic concentration of bilirubin.^[^ ^10^, ^11^ ^]^ Neurotoxic damage has been associated with bilirubin concentrations exceeding 40 mg dL^−1^.^[^ ^13^, ^47^ ^]^ However, bilirubin nanoparticles, such as BX‐001N, contain a small amount of bilirubin, which can be oxidized by ROS when released at lesion sites, thereby minimizing bilirubin toxicity.^[^ ^13^ ^]^ Biodistribution studies revealed that the BX‐001N primarily localized in the liver, where it is was excreted into the intestine, following the typical enterohepatic metabolic pathway.^[^ ^48^ ^]^ Importantly, it did not cross the blood‐brain barrier or accumulate in the brain, avoiding a risk for neurologic complications. BX‐001N showed significantly increased localization in the kidney after renal IRI. This enhanced localization likely resulted from the passive entry of the long‐circulating BX‐001N into the inflamed kidney, where leaky blood vessels enable tissue infiltration.^[^ ^49^, ^50^ ^]^

The renoprotective effects and adequate safety profile of BX‐001N compared to BR in this study, support BX‐001N as a potential new therapeutic agent for renal IRI, a condition currently lacking effective prevention or treatment beyond supportive care. Our regimen to administer BX‐001N both before and after IRI is applicable to predictable IRI related to transplantation and major vascular surgery. However, in cases of IRI related to shock, which are unpredictable, we may lose an opportunity to inject BX‐001N prior to IRI. These promising findings from rodent models should be validated in large animal models or clinical trials. We believe that further studies assessing the efficacy and safety of BX‐001N for IRI in kidney transplantation models will expand the indications of BX‐001N to cold IRI.

In conclusion, BX‐001N is the first GMP‐grade, synthetic bilirubin‐based nanomedicine. It effectively attenuates acute renal injury, facilitates subacute renal recovery, and suppresses chronic fibrosis after renal IRI via anti‐oxidant and immune‐modulating activity. Therefore, BX‐001N, with its renoprotective effects and adequate safety profile, is a promising new therapeutic agent for renal IRI.

## 4 Experimental Section

### Preparation of BX‐001N

BX‐001N was prepared using a flow chemistry method. Briefly, PEGylated bilirubin 3α was dissolved in a water‐miscible organic solvent and added dropwise into an aqueous phase while continuously stirring the mixture using a magnetic bar. The amphiphilic PEGylated bilirubin 3α monomers self‐assembled and spontaneously formed uniform PEGylated bilirubin 3α nanoparticles (BX‐001N). The freshly prepared BX‐001N® solution was further filtered using a 0.2 µm PVDF filter (sterilization for in vivo studies). Morphology analysis was performed by loading 10 µL of BX‐001N sample onto a coated Ni grid and staining with 5% uranyl acetate, before observing through a TEM.

### Measuring Intracellular and Extracellular ROS Levels and In Vitro Cytoprotective Effects

Human leukemia (HL‐60; KCLB, 10 240, Korea) and murine macrophage (RAW264.7; KCLB, 4007, Korea) cell lines were used to assess the in vitro effects of BX‐001N on oxidative stress and inflammation. HL‐60 cells were cultured with dimethyl sulfoxide (DMSO; Sigma‐Aldrich, USA) to induce the differentiation into neutrophil‐like cells. Subsequently, the dHL‐60 cells were treated with BR or BX‐001N with phorbol 12‐myristate 13‐acetate (PMA; Sigma‐Aldrich). The RAW264.7 cells were also treated with BR or BX‐001N and stimulated with lipopolysaccharide (LPS; Sigma‐Aldrich) and interferon‐gamma (IFN‐γ; R&D system, USA). The stimulated cells were treated with DCF‐DA (Cayman, 85 155, USA) or a fluorescein‐conjugated iNOS antibody (BD Bioscience, 610 331, USA).

The human proximal tubular cell line (HK‐2; KCLB, 22 190, Korea) was treated with different concentrations of BX‐001N or BR and 100 µm of H_2_O_2_ (Sigma‐Aldrich) for 24 h. The cell viability was examined using a WST‐8 cell viability assay kit (Biomax, Korea). The evaluation of extracellular and intracellular ROS scavenging efficacy in H₂O₂‐stimulated HK‐2 cells or HK‐2 cells under the hypoxia‐reoxygenation system is described in Supplementary Methods.

### Measuring Myeloperoxidase Activity and Elastase Activity

The differentiated HL‐60 cells were treated with PMA and BX‐001N. The MPO levels in the supernatants were measured using an MPO activity colorimetric assay kit (Cayman). For NET measurements, the differentiated HL‐60 cells were treated with PMA and BX‐001N. NETosis‐released neutrophil elastase in the supernatant was measured using a NETosis assay kit (Cayman).

### Animals and Renal Ischemia/Reperfusion Injury Models

Seven to eight‐week‐old male C57BL/6J mice (B6, SLC Inc., Japan) were used in all experiments. Bilateral renal IRI was induced by clamping both renal pedicles for 27 min.^[^ ^51^, ^52^ ^]^ For the renal fibrosis experiments, unilateral IRI was induced by clamping the left renal pedicle for 30 min. The mice were intravenously injected with either BX‐001N or PBS 1 h before and 1.5 h after IRI. The BR was intraperitoneally administered to mice. Serum creatinine and BUN levels were determined using QuantiChrom creatinine and urea assay kits (Bio‐Assay Systems, USA), respectively. The Institutional Animal Care and Use Committee of the Yonsei University Health System approved all experimental procedures (IACUC 2021‐0269, 2021.11.12). All experiments adhered to the NIH Guide for the Care and Use of Laboratory Animals.

### In Vivo Biodistribution of BX‐001N

The fluorescent Dil‐labeled BX‐001N were injected intravenously into mice 1 h before and 1.5 h after renal IRI or sham operation to assess the tissue distribution of BX‐001N in vivo (Figure 1B). The organs, including the brain, heart, lungs, liver, intestine, spleen, and kidneys, were excised 24 h after injection and imaged using an IVIS Spectrum Imaging System (PerkinElmer, USA).^[^ ^12^ ^]^

### Flow Cytometric Analysis and Real‐Time Polymerase Chain Reaction

Fluorochrome‐labeled antibodies and the primer sequences are listed in Tables S1 and S2 (Supporting Information), respectively.

### Measurement of Cytokines, Chemokine, and Renal ROS Activity

The levels of cytokines or chemokines were measured using enzyme‐linked immunosorbent assay kits (Table S1, Supporting Information). The oxidative biochemical parameters, including MDA (Abcam, UK), SOD activity (Cayman), and glutathione (Abcam) in kidney tissues, were measured using specific assay kits (Table S1, Supporting Information).

### Western Blotting

Protein expressions of fibronectin, type IV collagen, Nrf2, HO‐1, iNOS, Nox2, and β‐actin were measured (Table S1, Supporting Information).

### Renal Histology

The tubular injury score was assessed using periodic acid Schiff (PAS)‐stained kidney sections with grades 1–4.^[^ ^51^ ^]^ The renal fibrosis was assessed using Masson's trichrome staining. TUNEL staining (Roche Diagnostics, Switzerland), along with cleaved caspase‐3, bax, cytochrome c, and bcl‐2 were employed to evaluate the renal tubular apoptosis. Ki67 staining (Thermo Fisher Scientific) was used to assess renal tubular regeneration, respectively. The immunohistochemical staining for αSMA and nitrotyrosine was performed to evaluate the renal fibrosis and ROS‐induced tyrosine nitration, respectively. To assess the superoxide production, frozen kidney tissues were stained with DHE. All histological analyses were performed by two independent researchers who were blinded to the treatment groups.

### Statistical Analysis

The data were presented as mean with SEM. All samples in each group were displayed as individual dots in dot plots. The comparisons between groups were conducted using two‐sided Student's *t*‐tests for normally distributed data or Mann–Whitney test for non‐normally distributed data. Normal distribution was tested using the Shapiro–Wilk test. Statistical significance was set at *p* < 0.05. All analyses were performed using GraphPad Prism (version 10.10; GraphPad Software, USA).

## Conflict of Interest

H.K., S. Jo, D.S., S.M.A., and M.L.K. are employees of Bilix Co., Ltd., which provided BX‐001N for this research. S. Jon is a cofounder and scientific advisory board member of Bilix Co., Ltd. J.Y. received a grant from Bilix Co., Ltd.

## Author Contributions

J.J.Y. and H.K. equally contributed to this work. J.J.Y. and H.K. participated in data curation, methodology, formal analysis, and original draft manuscript; B.K., H.P., J.Y.J., T.K., W.B.L., D.K., S.J., D.S., and S.M.A. participated in data curation and methodology; M.L.K. participated in conceptualization and fund acquisition; S.J. participated in conceptualization and formal analysis; J.Y. participated in conceptualization, formal analysis, supervision, and editing manuscript. All authors approved the final version of the manuscript.

## Supporting information



Supporting Information

## Data Availability

All data are included in the manuscript or supporting Information. Any additional information will be available upon request.
