# Supplementary material for: Synthetic Bilirubin‐Based Nanomedicine Protects Against Renal Ischemia/Reperfusion Injury Through Antioxidant and Immune‐Modulating Activity
Source: Adv Healthc Mater. 2025 Jan 23;14(7):2403846. doi: 10.1002/adhm.202403846 (PMC11912105; doi:10.1002/adhm.202403846)
Supplement: Supplementary file 1 — Supporting Information [file ADHM-14-0-s001.docx]

Supporting Information

Synthetic Bilirubin-Based Nanomedicine Protects against Renal Ischemia/Reperfusion Injury through Antioxidant and Immune-Modulating Activity

Ji-Jing Yan, Hyunjin Kim, Bomin Kim, Honglin Piao, Joon Young Jang, Tae Kyeom Kang, Wook-Bin Lee, Dohyeon Kim, Seunghyun Jo, Duckhyang Shin, Sharif MD Abuzar, Myung L Kim, Jaeseok Yang, Sangyong Jon

Supplementary Methods

**Table S1.** Reagent information

**Table S2.** Primer sequences used for the study

**Figure S1.** Cytoprotective effects of BX-001N on renal tubular cells against oxidative stress

**Figure S2.** Renoprotective effects of different doses of BX-001N against renal IRI

**Figure S3.** Immunohistochemical staining for apoptosis-related molecules

**Figure S4.** Full-length Western blot images

**Supplementary Methods.**

*Preparation of BX-001N*

For *in vitro* experiments, lyophilized BX-001N was reconstituted in phosphate-buffered saline (PBS) to prepare a stock solution at a concentration of 20 mM, which was further diluted with PBS to produce 1,000 μM and 500 μM working solutions. For the formation of micelle-like nanoparticles in an aqueous buffer, the compound was thoroughly dissolved in PBS for 2–3 min by vortexing. All preparations were conducted in amber tubes to prevent light-induced degradation. To prepare free bilirubin (BR), 10 mg of bilirubin was suspended in 0.95 mL of 0.1 M sodium hydroxide (NaOH, pH 13.3). Subsequently, 50 μL of 1 M hydrochloric acid (HCl) was added to adjust the pH to 8.5, resulting in a 20 mM stock solution. This stock solution was further diluted using a 0.1 M NaOH:1 M HCl buffer (19:1, v/v) to prepare 10× working solutions at concentrations of 1,500 μM, 1,000 μM, and 500 μM. The prepared 10× working solutions of test compounds (BX-001N and BR) were diluted with the culture media at a 1:10 dilution ratio to achieve final concentrations ranging from 50 to 150 μM.

*Measuring intracellular reactive oxygen species (ROS) levels of human immune cells*

Human leukemia (HL-60; KCLB, 10240, Korea) and murine macrophage (RAW264.7; KCLB, 4007, Korea) cell lines were used to assess the *in vitro* effects of BX-001N on oxidative stress and inflammation. HL-60 cells were cultured with dimethyl sulfoxide (DMSO; Sigma-Aldrich, USA) for 72 h to induce the differentiation into neutrophil-like cells. Subsequently, the differentiated HL-60 (dHL-60) cells were treated with BR or BX-001N with phorbol 12-myristate 13-acetate (PMA; Sigma-Aldrich) for 8 h. The RAW264.7 cells were also treated with bilirubin or BX-001N for 4 h and stimulated with lipopolysaccharide (LPS; Sigma-Aldrich) and interferon-gamma (IFN-γ; R&D system, USA) for 24 h. The stimulated cells were treated with an oxidative-sensitive fluorescent probe (2′,7′-dichlorodihydrofluorescein diacetate [DCF-DA], 20–25 μM; Cayman, USA) for 45 min. To compare inducible nitric oxide synthase (iNOS) levels, the RAW264.7 cells were fixed, permeabilized with 2% paraformaldehyde for 10 min, and stained with a fluorescein-conjugated iNOS antibody (BD Bioscience, 610331, USA). The fluorescence intensity of the cells was measured using a multimode microplate reader.

*Measuring extracellular and intracellular ROS levels of human renal tubular cells*

To evaluate the ROS scavenging potency of BX-001N at the cellular level, oxidative stress was induced in HK-2 cells by treating them with H₂O₂ (Sigma-Aldrich, MAK-165B, USA). HK-2 cells were seeded into 96-well plates (SPL, 33396, South Korea) and incubated for 24 h. Subsequently, the cells were treated with varying concentrations of bilirubin or BX-001N (0–250 μM) and 20 μM of H₂O₂ for 24 h. After incubation, the extracellular ROS levels (H₂O₂ and O₂⁻) were measured by collecting the supernatant. H₂O₂ and O₂⁻ levels were measured using the Amersham ECL Plus kit (Cytiva, RPN2232, USA) and the Superoxide Anion Scavenging Capacity Assay Kit (mybiosource, MBS9718966, USA), respectively. To measure intracellular ROS levels, adherent cells were treated with 25 μM of DCF-DA (Cayman, 85155, USA) and incubated. Fluorescence intensity was measured using a multimode microplate reader. The obtained values were plotted as [inhibitor] vs. response, and the EC_50_ of test compounds for ROS scavenging in HK-2 cells was calculated using the variable slope equation.

A hypoxic cell culture model was established using a hypoxic cell culture kit (nBIONIX-3, Bulldog Bio, USA) to mimic the ischemia-reperfusion injury environment *in vitro*. HK-2 cells were seeded into 96-well plates and incubated for 24 h. Subsequently, the medium was replaced with serum and glucose-free RPMI 1640 medium (Gibco; 11879-020, USA), and test compounds (BX-001N or bilirubin) were added. Hypoxia was induced by sealing the plates with pouch clips to maintain an oxygen-depleted environment. After 4 h, the clips were removed to restore the oxygen level to 20%, and the medium was replaced with a complete RPMI 1640 medium, simulating the reoxygenation condition. At this point, the test compounds were re-administered. After 20 h of reoxygenation, the supernatant was collected for measuring extracellular ROS (H₂O₂ and O₂⁻) levels using the methods described above. The adherent cells were used to measure intracellular ROS levels with DCF-DA as previously described.

*Animal experiments*

No criteria existed for including or excluding animals during the experiments. No randomization was used to allocate mice to the PBS, BR, and BX-001N groups. All mice were maintained under specific pathogen-free conditions. The mouse cages in three groups were kept on the same levels of the same rack. All animals were housed and received humane care according to the Principles of Laboratory Animal Care as formulated by the National Society for Medical Research. Tramadol (0.25 mg/kg; Jeil Pharmaceutical, Seoul, Republic of Korea) was subcutaneously administrated immediately after a skin suture to relieve pain. The experiments were conducted by researchers who were not blinded to the treatment groups; however, the data analysis including outcome analysis was done by researchers who were blinded to the treatment groups.

*Flow cytometric analysis*

The kidneys were disrupted using a Stomacher 80 Biomaster (Sewart, Worthing, United Kingdom), and renal leukocytes were isolated using a Percoll gradient (GE Healthcare Bio-Sciences, Uppsala, Sweden). The isolated cells were stained with fluorochrome-labeled antibodies (Table S1) and analyzed using an Attune NxT flow cytometer (Thermo Fisher Scientific).

*Real-time polymerase chain reaction*

Total RNA was extracted from homogenized renal tissues and real-time reverse transcription polymerase chain reaction analysis was performed using QuantStudio (v.3.0; Thermo Fisher Scientific). The relative mRNA expression of each gene was determined using the -ΔΔCt calculation method with **glyceraldehyde 3-phosphate dehydrogenase** (GAPDH) as the internal control gene. The primer sequences are listed in Table S2.

*Measurement of cytokines and chemokines*

The levels of cytokines or chemokines were measured using enzyme-linked immunosorbent assay kits (Table S1). The kidney tissue was homogenized in RIPA lysis buffer containing a protease inhibitor cocktail (Thermo Fisher Scientific). The expression values were normalized to protein levels in renal tissues.

*Western blotting*

The primary antibodies towards fibronectin, type IV collagen, nuclear factor erythroid 2-related factor (Nrf), heme oxygenase 1 (HO-1), iNOS, nicotinamide adenine dinucleotide phosphate oxidase 2 (Nox2), and β-actin were used (Table S1). Subsequently, the membranes were incubated with appropriate secondary antibodies conjugated with horseradish peroxidase (1:5000 dilution). The band intensity was quantified using ImageJ software.

*Renal histology*

The kidneys were fixed in 10% neutral-buffered formalin for 36 h before paraffin embedding. Histological assessment was performed on periodic acid Schiff (PAS)-stained sections. For immunohistochemical analysis, sections were dewaxed, rehydrated and subjected to microwave-based antigen retrieval, followed by blocking with hydrogen peroxide. Sections were then probed with primary antibodies (Table 1S). Subsequently, secondary antibody staining was performed using the rabbit Polink-2 HRP kit (OriGene Technologies, Rockville, USA) and DAB staining (Vector Labs), followed by counterstaining with hematoxylin. Images were captured with using a ZEISS Axioscan7 Microscope Slide Scanner (Carl Zeiss Microscopy Deutschland GmbH, Oberkochen, Germany). Cell counting and analysis of positive cells and area were performed using ZEISS ZEN 3.4 image analysis software, counting four high-power fields (200x) per section. The confocal imaging was performed using an LSM710 confocal laser microscopy (Carl Zeiss, Meditec, Germany). Two independent researchers who were blinded to the treatment groups performed all histological analyses.

*Statistical analysis*

Normal distribution of data was tested using the Shapiro–Wilk test. When the data did not follow a normal distribution, the Mann–Whitney test was employed and the data were presented as the median with the interquartile range.

**Table S1.** Reagent information.

| **Reagents** | **Identifier** | **Source (company)** |
| --- | --- | --- |
| Antibodies | | |
| anti-αSMA | Cat#ab124964, Clone: EPR5368, Use: WB, IHC | Abcam |
| anti-β-Actin | Cat#ab115777, Clone: SP124 Use: WB | Abcam |
| anti-Bax | Cat#Bsm-52316, Clone: 3C3 Use: IHC | Bioss |
| anti-Bcl-2 | Cat#ab182858, Clone: EPR17509 Use: IHC | Abcam |
| anti-CD11b-PE | Cat#553311, Clone: M1/70, Use: FACS | BD Biosciences |
| anti-CD19 PE-cy7 | Cat#115520, Clone: 6D5, Use: FACS | BioLegend |
| anti-CD3-APC | Cat#17-0032, Clone: 17A2, Use: FACS | Thermo Fisher Scientific |
| anti-CD45-V421 | Cat#103134, Clone: 30-F11, Use: FACS | BioLegend |
| anti-cleaved caspase-3 | Cat#9664, Clone: Asp175 Use: IHC | Cell signaling tecnology |
| anti-Col-IV | Cat#ab6586, polyclonal, Use: WB | Abcam |
| Anti-cytochrome c | Cat#ab133504, EPR1327, Use: IHC | Abcam |
| anti-F4/80-FITC | Cat#11-4801, Clone: BM8, Use: FACS. | Thermo Fisher Scientific |
| anti-Fibronectin | Cat#ab45688, Clone: F14, Use: WB | Abcam |
| anti-Gr-1-APC | Cat#17-5931, Clone: RB6-8C5, Use: FACS | Thermo Fisher Scientific |
| Anti-HO-1 | Cat#ab13248, Clone: Ho-1-1, Use: WB | Abcam |
| anti-Ki67 | Cat#ab16667, Clone: SP6, Use: IHC | Abcam |
| anti-iNOS | Cat#610331, use: IF | BD bioscience |
| anti-iNOS | Cat#2982, Polyclonal, Use: WB | Cell signaling |
| anti-Nox2 | Cat#ab129068, Clone: EPR6991, Use: WB | Abcam |
| anti-nitrotyrosine | Cat#A-21285. Use: IHC | Thermo Fisher Scientific |
| anti-Nrf2 | Cat#PA5-27882, Use: WB | Thermo Fisher Scientific |
| Chemical or recombinant protein | | |
| 7-AAD | Cat#00-6993-50, Use: FACS | Thermo Fisher Scientific |
| Dil | Cat#D282 | Thermo Fisher Scientific |
| DCF-DA | Cat#85155 | Cayman |
| Dihydroethidium (DHE) | Cat#D11347 | Thermo Fisher Scientific |
| Dihydrochloride | Cat#62247 | Thermo Fisher Scientific |
| Hoechst 33342 | Cat#R37605 | Thermo Fisher Scientific |
| Hydrogen peroxide | Cat#MAC-165B | Merk |
| Recombinant mouse IFN-γ | Cat#485-MI | R&D system |
| Lipopolysaccharide (LPS) | Cat#L2880 | Merk |
| Phorbol 12-myristate 13-acetate | Cat#P8138 | Merk |
| Assay kits |  |  |
| MPO activity colorimetric assay kit | Cat#600620 | Cayman |
| NETosis assay kit | Cat#600610 | Cayman |
| Glutathione assay kit | Cat#ab239727 | Abcam |
| Human IL-8 ELISA kit | Cat#SM6000B | R&D system |
| Malondialdehyde ELISA kit | Cat#MBS269473 | MyBioSource |
| Human TNF-α ELISA kit | SSTA00E | R&D system |
| Human IL-1β ELISA kit | SLB50 | R&D system |
| Human IL-8 ELISA kit | DY208-05 | R&D system |
| Mouse CXCL1 ELISA kit | Cat#447504 | BioLegend |
| Mouse CXCL2 ELISA Kit | Cat#900-K152 | Pepro Tech |
| Mouse IFN-γ ELISA kit | Cat#430801 | BioLegend |
| Mouse IL-10 ELISA kit | Cat#431411 | BioLegend |
| Mouse MCP-1 ELISA kit | Cat#446207 | BioLegend |
| Mouse TNF-α ELISA kit | Cat#430907 | BioLegend |
| Mouse TNF-α ELISA kit | Cat#430907, SMTA00B | BioLegend, R&D system |
| Mouse IL-1β ELISA kit | DY401-05 | R&D system |
| Polink-2 HRP rabbit DAB kit | D39-18 | OriGene |
| QuantiChrom™ creatinine assay Kit | Cat#DICT-500 | Bioassay Systems |
| QuantiChrom™ urea assay Kit | Cat#DIUR-100 | Bioassay Systems |
| Superoxide dismutase assay kit | Cat#ab239727 | Cayman |
| TUNEL assay kit | Cat#ab206386 | Abcam |
| WST-8 cell viability assay kit | Cat#QM2500 | Biomax |

7-AAD, 7-aminoactinomycin D; APC, allophycocyanin; αSMA, α-smooth muscle actin; Col-IV, type IV collagen; Cxcl, chemokine (C-X-C motif) ligand; DCF-DA, 2′,7′-dichlorodihydrofluorescein diacetate; DHE, dihydroethidium; Dil, 1,1'-dioctadecyl-3,3,3',3'-tetramethylindocarbocyanine perchlorate; ELISA, enzyme-linked immunosorbent assay; FACS, fluorescence-activated cell sorting; FITC, fluorescein; Foxp3, forkhead box P3; HO-1, Heme oxygenase 1;  IFN-γ, Interferon-γ; IF, immunofluorescence staining; IHC, immunohistochemical staining; IL, interleukin; iNOS, inducible nitric oxide synthase; MCP-1, Monocyte Chemotactic Protein 1; Nox2, nicotinamide adenine dinucleotide phosphate oxidase 2; MPO, Myeloperoxidase; NET, neutrophil extracellular traps; Nrf2, Nuclear factor erythroid-2-related factor 2; PE, phycoerythrin; TNF-α, Tumor necrosis factor; TUNEL, terminal deoxynucleotidyl transferase dUTP nick-end labeling; WB, western blot.

**Table S2.** Primer sequences used for the study.

| **Gene** | **Gene Bank ID** | **Primer sequence (5’-3’)** |
| --- | --- | --- |
| *Col4a1* | *NM_009931.2* | F: GCTCTGGCTGTGGAAAATGTG  R: GTTCTCCAGCATCACCCTTTTG |
| *Cxcl1* | *NM_008176.3* | F: ACTCAAGAATGGTCGCGAGG  R: GTGCCATCAGAGCAGTCTGT |
| *Cxcl2* | *NM_009140.2* | F: CACTCTCAAGGGCGGTCAAA  R: GGTTCTTCCGTTGAGGGACA |
| *Fn1* | *NM_010233* | F: TTGGTGATGTGTGAAGGCTC  R: ACCTCTGCAGACCTACCCAG |
| *Gapdh* | *NM_008084* | F: CAGAGGCGCATGAAGCTAATG  R: CCTGGATCTTCCTCACTTGCT |
| *HO-1* | *NM_010442.2* | F: TCTGGTATGGGCCTCACTGG  R: GTCACCCAGGTAGCGGGTAT |
| *IFN-γ* | *NM_008337* | F: GAGCTCATTGAATGCTTGGC  R: GCGTCATTGAATCACACCTG |
| *Il10* | *NM_010548* | F: GGTGAGAAGCTGAAGACCCT  R: TGTCTAGGTCCTGGAGTCCA |
| *iNos* | *NM_010927.4* | F: GAAACTTCTCAGCCACCTTGG  R: TCCAAATCCAACGTTCTCCGT |
| *Mcp1* | *NM_011333* | F: TTCCACAACCACCTCAAGCACTTC  R: TTAAGGCATCACAGTCCGAGTCAC |
| *Nox2* | *NM_007807.5* | F: ACAACCCTCCCTGTCTAGGTA  R: GCCTTCGGTGATGTGCTTTAC |
| *Nrf2* | *NM_010902.5* | F: GCTGCTCGGACTAGCCATTG  R: TCAAATCCATGTCCTGCTGGG |
| *Tnfa* | *NM_013693* | F: ATGTCCATTCCTGAGTTCTG  R: AATCTGGAAAGGTCTGAAGG |

*Col-IV*, type IV collagen; *Cxcl*, chemokine (C-X-C motif) ligand; **F,** forward; *Fn1*, fibronectin; *Gapdh*, **Glyceraldehyde 3-phosphate dehydrogenase;** *HO-1*, Heme oxygenase 1;*IFN-γ*, Interferon-γ; *IL*, interleukin; *iNOS*, inducible nitric oxide synthase; *Mcp-1*, Monocyte Chemotactic Protein 1; *Nox2*, Nicotinamide adenine dinucleotide phosphate oxidase 2; *Nrf2*, Nuclear factor erythroid-2-related factor 2; R, reverse; *TNF-α*, tumor necrosis factor-α

**

**

**Figure S1.** Cytoprotective effects of BX-001N on renal tubular cells against oxidative stress. A) HK-2 cells were stimulated by H_2_O_2_ with various concentrations of BX-001N or BR. Cellular viability of HK-2 cells was expressed as a relative proportion compared to that of the negative control group without H_2_O_2_ stimulation. N =6 for each group. B) Evaluation of extracellular H₂O₂ scavenging efficacy in H₂O₂-stimulated HK-2 cells. C) Evaluation of extracellular H₂O₂ scavenging efficacy in HK-2 cells under the hypoxia-reoxygenation system. D) Evaluation of extracellular O₂^-^ scavenging efficacy in H₂O₂-stimulated HK-2 cells. E) Evaluation of extracellular O₂^-^ scavenging efficacy in HK-2 cells under the hypoxia-reoxygenation system. F) Evaluation of intracellular ROS scavenging efficacy in H₂O₂-stimulated HK-2 cells using DCF-DA staining. G) Evaluation of extracellular ROS scavenging efficacy in HK-2 cells under the hypoxia-reoxygenation system using DCF-DA staining. Lines indicate the mean and SEM, respectively. The obtained values in Figure S1B, D, and F were plotted as [inhibitor] vs. response, and the EC_50_ of test compounds for ROS scavenging in HK-2 cells was calculated using the variable slope equation. **P* < 0.05, ***P* < 0.01 compared to the positive control group with H_2_O_2_ stimulation or hypoxia-reoxygenation but without BX-001N (Student’s t-test or Mann-Whitney test). ^#^*P* < 0.05, ^##^*P* < 0.01 in comparison between the BX-001N and BR groups (Student’s t-test).

BR, free bilirubin group; DCF-DA, 2′,7′-dichlorodihydrofluorescein diacetate; EC_50_, half maximal effective concentration; H & R, hypoxia-reoxygenation; ROS, reactive oxygen species; SEM, standard error of the mean.

**
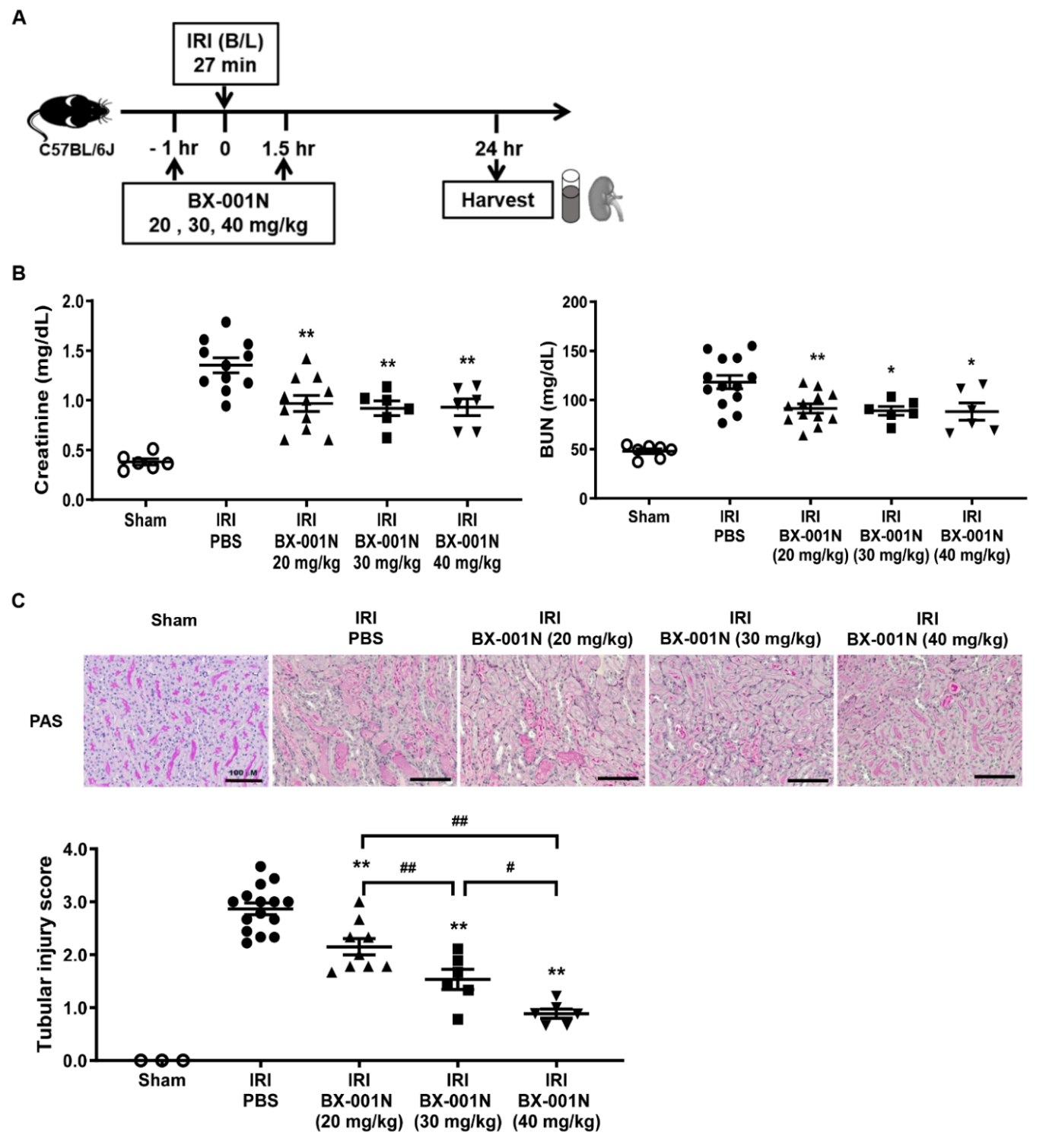
**

**Figure S2.** Renoprotective effects of different doses of BX-001N against renal IRI. A) PBS or BX-001N (20, 30, and 40 mg/kg) was administered to mice 1 h prior to and 1.5 h following renal IRI. Kidneys along with blood samples were procured on day 1 after IRI. B) Blood levels of creatinine and BUN. C) Renal tissue injury scores based on PAS staining. Scale bars, 100 μm. Magnification, 200×. All samples in each group are displayed as individual dots, and lines and whiskers in dot plots indicate the mean and SEM, respectively. **P* < 0.05, ***P* < 0.01 compared to the PBS group. ^#^*P* < 0.05, ^##^*P* < 0.01 in comparison between different dose groups of BX-001N (Student’s t-test). BUN, blood urea nitrogen; IRI, ischemia-reperfusion injury; PAS, periodic acid–Schiff; PBS, phosphate-buffered saline; SEM, standard error of the mean.


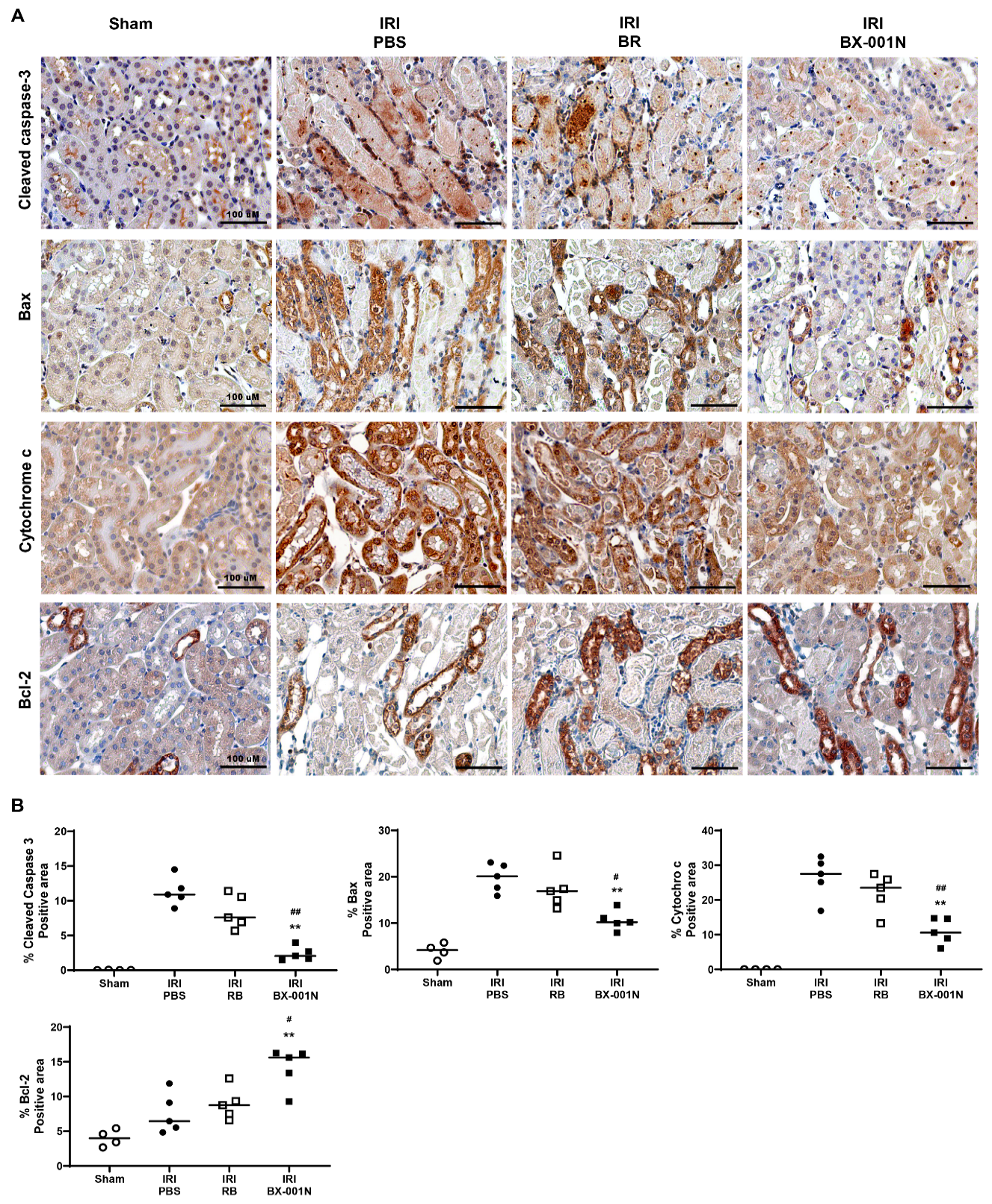


**Figure S3.** Immunohistochemical staining for apoptosis-related molecules. A) Staining images of cleaved caspase-3, bax, cytochrome c, and bcl-2. Scale bars, 100 μm. Magnification, 200×. B) Renal tubular expression levels of pro-apoptotic and anti-apoptotic molecules. All samples in each group are displayed as individual dots, and lines and whiskers in dot plots indicate the mean and SEM, respectively. ***P* < 0.01 compared to the PBS group. ^#^*P* < 0.05, ^##^*P* < 0.01 in comparison between the BX-001N and BR groups (Student’s t-test). BR, free bilirubin group; IRI, ischemia-reperfusion injury; PBS, phosphate-buffered saline; SEM, standard error of the mean.





**Figure S4.** Full-length Western blot images. A) Full Western blot images for Figure 7f (fibronectin, type IV collagen, and β-actin). B) Full Western blot images for Figure 9b (Nox2, iNOS, Nrf2, HO-1, and β-actin). Col-IV, type IV collagen; HO-1, heme oxygenase-1; iNOS, inducible nitric oxide synthase; Nox, nicotinamide adenine dinucleotide phosphate oxidase; Nrf2, nuclear factor erythroid-2-related factor 2.
